# Three Colleges, Etc.

**Published:** 1881-08-20

**Authors:** 


*There are three College advertisements* in our Journal which our readers are requested to examine. They are the JEFFERSON, Philadelphia; MEDICAL COLLEGE OF LOUISIANA, New Orleans, and the SOUTHERN MEDICAL COLLEGE, Atlanta, Ga.

